# Environmental impact ramifications of replacing added sugar with non-nutritive sweeteners in a high-calorie Dutch diet: the SWEET project

**DOI:** 10.1007/s00394-026-04127-z

**Published:** 2026-10-07

**Authors:** J. Suckling, A. C. Grasso, E. Koukouna, J. M. Groen, M. Tyszler, S. Morse, M. Raats, S. B. Astley, J. C. G. Halford, J. A. Harrold, A. Raben, J. Scholten, C. Scott, R. Murphy

**Affiliations:** 1 Centre for Environment and Sustainability, University of Surrey, Guildford, UK; 2 Mérieux NutriSciences | Blonk, Rotterdam, The Netherlands; 3 School of Psychology, University of Surrey, Guildford, UK; 4 EuroFIR AISBL, Brussels, Belgium; 5 School of Psychology, University of Leeds, Leeds, UK; 6 Department of Psychology, University of Liverpool, Liverpool, UK; 7 Department of Nutrition, Exercise and Sports, Clinical Research, University of Copenhagen, Copenhagen University Hospital – Steno Diabetes Center Copenhagen, Copenhagen, Herlev, Denmark; 8 Cargill, B.V, Amsterdam, The Netherlands; 9 Institute for Sustainability, University of Surrey, Guildford, UK

**Keywords:** Life cycle assessment, Non-nutritive sweeteners, Whole diet, Added sugar, Nutrition

## Abstract

**Purpose:**

Growing concern about excess added sugar intake and associated adverse health effects has increased interest in non-nutritive sweeteners (NNS) as low-calorie alternatives that preserve sweetness. Previous studies focus on health effects of replacing added sugar, or environmental impacts of NNS alone. However, none evaluate the environmental impact ramifications of replacing added sugar with NNS in a whole dietary setting. This study addresses that gap.

**Methods:**

The baseline diet reflected an average diet of Dutch adults consuming > 2200 kcal/day (mean 2866 kcal/day). Added sugar (54 g/day), identified from recipe data, was replaced with steviol glycosides and a bulking agent (e.g., sorbitol) to create the NNS diet. Both diets were linked to life cycle assessment data and optimized separately for nutritional adequacy and the 2023 IPCC 1.5 °C target of 2.04 kgCO_2_-eq/day.

**Results:**

Both diets had equivalent global warming potential (GWP, 4.18 kgCO_2_-eq/day), but the NNS diet used 11% more water (compared to 1.19 × 10^− 1^ m^3^/day of baseline diet). When optimized for nutritional adequacy, GWP for both diets reduced 13%, and water consumption increased 3–9%. When optimized for nutrient adequacy and emissions targets, water consumption decreased by 11–15% across both diets.

**Conclusion:**

These results highlight an opportunity for policymakers and consumers to focus on the health benefits of added sugar reduction rather than environmental trade-offs. Both dietary patterns can be further improved regarding nutritional and climate goals, suggesting that balanced, low-emission diets are attainable across different sweetness strategies. The study identifies methodological challenges in modeling realistic dietary substitutions and provides guidance for future research.

**Supplementary Information:**

The online version contains supplementary material available at https://doi.org/10.1007/s00394-026-04127-z.

## Introduction

There is increasing concern regarding over-consumption of added sugars in diets, with many people consuming more than the recommended 10% total calorie intake from free sugars [1]. For example, in the Dutch population, free sugars comprised 14% total calorie intake [2], and 12.4% in UK diets [3]. Such over-consumption is linked to adverse health conditions such as obesity, diabetes [4], and tooth decay [5]. Free sugars include naturally occurring sugars in honey, syrups and unsweetened fruit juices as well as sugars added to food or drinks. Added sugars may be replaced by other ingredients. For instance, high-intensity non-nutritive sweeteners (NNS) are proposed as a means of providing sweet taste and many fewer calories, without the adverse health side-effects [6]. Examples of widely used NNS are aspartame, sucralose or steviol glycosides, each with a sweet taste many hundreds of times more potent than sucrose (respectively approx. 200, 650, and 50–450 times sweeter when used in yoghurt) [7]. For NNS to be ratified as safe for consumption, there is by necessity, large bodies of research into their safety e.g., [8]. Similarly, there is an increasing body of evidence relating to the health effects of consuming them instead of added sugar [9], often in the context of a dietary setting. However, research into the environmental impact ramifications of replacing added sugar with NNS, in any setting, is less well developed.

Most environmental impact research on NNS has focused on steviol glycosides [10–13]. There are also studies on aspartame or neotame [14], sucralose [15], and the sweetness enhancer, thaumatin [16]. Finally, there are data for stevioside and aspartame in the World Food LCA Database (WFLDB) [17]. In general, studies show that NNS have lower environmental impact than sugar when providing an equivalent sweetness, primarily due to the much lower masses of NNS required to replace the equivalent sweetness (e.g., 5 g aspartame per 1 kg sugar). However, all these studies are only for the NNS in isolation. The authors are aware of only one environmental impact assessment of NNS used in context of foods, for drinks and yoghurts [18]. Further extending the research to a whole diet perspective is imperative if we are to understand any potential consequences of using NNS in place of added sugar at that larger scale [19]. At present, the authors are not aware of any such research, and this is a gap that needs to be filled. This work aims to start that process.

When replacing added sugar with NNS in foodstuffs, it is not always possible to directly replace one with the other. Greater-intensity sweetness of NNS means that a large mass of sugar can be replaced with a small mass of NNS e.g., [20, 21]. As a result, the greater bulk of sugar must also be replaced [22]. However, sugar does not only provide bulk and sweetness; it also serves other technical functions (e.g., texture, hydroscopic control, mouth feel). Therefore, other ingredients are required alongside NNS, for instance sugar alcohols (e.g., sorbitol, maltitol). These are hydrogenated sugars, and provide many of the technical functions, but with fewer calories per gram [23].

Environmental studies for sugar alcohols exist, but the literature is by no means complete. Studies relating to sorbitol are most numerous [24–27]. However, the authors are only aware of one relating to maltitol [24]. The authors are not aware of any studies for other sugar alcohols. The studies show that environmental impacts of sorbitol and maltitol tend to be greater than sugar on a like-for-like mass basis. This is because the original sugars (glucose for sorbitol and maltose for maltitol) require further processing to hydrogenate them, leading to more environmental impact. Sugar alcohols tend to have lower sweetness than sugar and, therefore, may need to be paired with an NNS to achieve the same sweetness for a given mass. In such instances, the NNS contributes only a small fraction of the combined environmental impact e.g., [12, 14].

In terms of reformulation, it is not always appropriate to use just one ingredient to replace added sugar in every type of food. Added sugars take the form of, for example, sucrose, high fructose corn syrups (HFCS), or glucose syrups. Multiple, different replacement ingredients may need to be identified. This is another gap in research, and one which many companies are exploring. However, at present, much of the research is proprietary, and formulation data are not readily accessible [22]. Therefore, more research is required into potential hurdles that must be overcome to understand the environmental impacts of replacing added sugar with NNS. This is a nuanced gap, and this research aims to highlight those hurdles, and provide clarity on potential avenues of research required to overcome them.

Understanding the implications of each food does little to highlight potential consequences of whole dietary change. There are numerous studies into the reduction of environmental impact by replacing certain food groups, for example meat [28]. However, the authors are not aware of any that have attempted to quantify environmental impact of replacing added sugar with NNS at the dietary level.

There are multiple gaps that this study aims to fill, and several challenges that it aims to highlight. The overarching purpose of the study was to understand the ramifications of replacing added sugar with NNS and other necessary ingredients, in a whole dietary setting. The study has the following objectives:

- Identify and replace added sugars in the context of a whole dietary setting.
- Quantify the environmental impacts of both the added sugar (baseline) and NNS diets.
- Understand consequences of trying to optimize the baseline diet to meet the calorie availability of the NNS diet.
- Understand whether either diet can be adjusted to meet sustainable dietary targets for 2030.
- Identify and catalogue the challenges of undertaking such a study, to aid in guidance for future research programs.

## Materials and methods

This study compares two diets with differing sources of sweetness (sugar or NNS). The study also utilizes optimization to explore shifts in diet composition required to meet nutritional requirements and reduced carbon footprint of the diets. The study used a combination of life cycle assessment approaches and dietary optimization Optimeal 3.0^®^ software (Mérieux NutriSciences | Blonk, Rotterdam, The Netherlands). Life cycle assessment is a systematic approach to quantifying environmental impact of product or services. The methodology is outlined in the ISO 14040:2006 [29] and ISO 14044 [30] standards. It provides a standardized approach to quantifying impact enabling comparison between different systems; in this case a diet sweetened with sugar and one sweetened with NNS and bulking agents.

### Current diet

Dietary data of Dutch adults aged 18–50 years were derived from the Dutch National Food Consumption Survey (DNFCS, 2019–2021) [31]. The DNFCS is a nation-wide survey of food consumption in the Dutch population across ages 1–79y. It covers consumption across various food groups and calories and nutrients ingested therein, use of dietary supplements, location and time of consumption, and which fraction of the population meet dietary recommendations. It forms part of a series of surveys that enable tracking of dietary changes over time. Data were collected from 602 adults, 18–50y by means of two 24-hour dietary recalls on two non-consecutive days. Body mass index (BMI) was calculated as self-reported body weight divided by height squared (kg/m^2^). Adults with a BMI *≥* 30 kg/m^2^ were considered obese (*N* = 107) and were initially the focus of this analysis. Due to the higher potential for underreporting of energy intake within this subset [32], and lower than expected average reported energy intake (2001 kcal/d, see Online Resource Table S1), it was decided instead to study a higher-energy consuming subset of adults with a calorie intake of > 2200 kcal/d. This threshold was set as a pragmatic cut-off to mitigate against underreporting bias, while seeking to preserve a balance sample of male and female participants. However, it is acknowledged that calorie intake is highly individual, and dependent on sex, age, body size, and physical activity levels. The resulting analytical sample included 143 respondents (72 men and 71 women) who consumed a diet providing > 2200 kcal/d and had a mean age of 38 *±* 9 y, mean body weight of 82.2 *±* 16.4 kg and a mean BMI of 26 *±* 4.8 kg/m^2^.

Consumption data for 825 foods, reported by adults consuming a high-energy diet, were linked to an extended version of the Dutch Food Composition Database NEVO v7.1 [33]. Due to limited environmental impact data, numbers of foods were reduced to 386 products by excluding foods contributing < 1% to total weight (in grams) and energy (in kcal) of the respective food group. Then, the weight of excluded foods were proportionally redistributed across included foods in each (sub)food group. Furthermore, foods such as meal replacers were excluded. GloboDiet Food group classifications were used, with the main food groups given in Online Resource, Table S5, and the sub-groups listed in (Table B1 [31]), .

### Life cycle assessment

Life cycle assessment (LCA) methodology was used to calculate global warming potential (GWP), land use, terrestrial acidification (TA), freshwater eutrophication (FEu), marine eutrophication (MEu), and water consumption (WC). These impact categories were chosen for their connections to agriculture and food production.

LCAs accounted for environmental impacts across the full supply chain (i.e., cultivation, post-harvest, processing, transport, distribution, packaging, preparation, and waste produced during the production process and after consumption). The LCAs were derived from Blonk Food Database [34], which contains LCA data for food products commonly consumed in the Netherlands and serves as the basis of the updated Dutch National Institute for Public Health and the Environment environmental impact of foods database [33]. The characterization method used was ReCiPe 2016 Midpoint (Hierarchist) [35]. Background life cycle inventory (LCI) data were derived from the Agri-footprint 6.3 database, which primarily includes agricultural commodities and feed materials rather than finished food products [36]. To enhance coverage, the database was expanded to include cultivation data for fruits and vegetables. These additional LCIs were developed following the same methodological principles outlined in the Agri-footprint 6.3 documentation [36]. Furthermore, system boundaries were extended to include food processing, distribution, retail, preparation and consumption to ensure consistency with the goal and scope of the food LCA database. About 42% of food items in the reduced diet had a direct match to foods in the LCA database. For food items with missing environmental impact data, environmental impacts of similar food products were used as proxies and, when a suitable proxy could not be identified, weighted averages of impacts of similar foods were used. This is approach is similar to previous studies [37].

In addition to the pre-existing LCA database foods, three other, new ingredients were necessary to complete anaylsis: steviol glycosides (specifically a glycoside mix containing at least 60% Rebaudioside A, with 95% purity, aka RA60); sorbitol; maltitol syrup. These were not available in the databases, and existing LCI data were not available in the detail required. Therefore, impact data were derived from studies conducted specifically for the research, as part of the SWEET project [38]. Full LCI of RA60 is available in [12], with summary environmental impact data per 1 kg RA60 given in Online Resource Table S2. In summary, the RA60 is produced from *Stevia rebaudiana* grown in Lamia, Greece, and extracted and purified near Riom, France. The GWP of the RA60 is 20.25 kgCO_2_-eq/kg. RA60 was chosen for this study over other variants of Reb A (e.g., RA80 or RA95) due to the lack of LCA data for these other two ingredients. Possible ramifications of this choice are discussed in Sect.  4.3.

For the bulking agents (sorbitol and a maltitol by-product syrup, aka maltitol-BPS), data were derived from process simulation using wheat starch. Specifics of the study are available in [24], but environmental impact data per 1 kg are available in Online Resource Table S3. Maltitol-BPS was used as a proxy for liquid sugars identified in the original food formulations. The GWP of the sorbitol is 1.61 kgCO_2_-eq/kg, and maltitol-BPS is 1.41 × 10^− 1^ kgCO_2_-eq/kg.

It should be noted that other sweeteners could have been used rather than RA60. However, it has also been shown that the choice of sweetener has little effect on final environmental impact of a foodstuff [18]. Furthermore, when mixed with a bulking agent, it is the bulking agent that contributes most to the environmental impact [24]. Therefore, it is assumed that the same is likely in this instance, but this is acknowledged as an area that needs future investigation.

### Nutritional data

#### Added sugar

The extended version of the Dutch Food Composition Database ‘NEVO’ v7.1 [33] contains data on more than 130 nutrients, but it does not contain values for added sugar. Instead, it gives total mono- and disaccharides present in the food, which are a combination of free and added sugars. Therefore, we used recipe data from the Blonk Food Database [34] LCIs to determine how much sugar is added to food and beverage products. For instance, the recipe used for 1 kg cake with butter included 275 g of beet sugar, so we deduced there was 27.5 g added sugar per 100 g of cake with butter. If the amount of added sugar deduced from the Blonk Food Database LCIs was greater than the total mono- and disaccharides of the food product derived from NEVO, we adjusted amounts of added sugar to equal that of total mono- and disaccharides in the latter. This was done because added sugars cannot be greater in mass than total of all mono- and di-saccharides as given in NEVO. For example, cake with butter was used as a proxy for Dutch butter cake (‘boterkoek’), which contains 25.4 g mono- and disaccharides per 100 g (less than 27.5 g). Therefore, we adjusted values so the amount of added sugar in boterkoek was 25.4 g per 100 g. This approach facilitates an estimation of added sugar despite the lack of more detailed data in NEVO. Mixed meals were considered as recipes, and ingredients were classified as per the food (sub)groups, as given in [31]. An adjustment had to be made for 44 out of 139 food products that had sugar as an ingredient in the corresponding LCI. Other sugars in scope included glucose syrup and HFCS.

#### Sweetener and bulking agents

In this study, we focused on RA60, meaning that it contains zero or very low amounts of carbohydrates and energy. Regardless, the three bulking agents in scope (i.e., sorbitol, maltitol-BPS and milk) do contain both carbohydrates and energy. Sorbitol and maltitol are polyols, or sugar alcohols with an energy content 260 kcal/100 g and 210 kcal/100 g, respectively [39]. We assumed a water content of 50% for maltitol-BPS, so the energy content used was 105 kcal/100 g. Lactose is a naturally occurring sugar in milk and has an energy content of 61 kcal/100 g.

### Sugar replacement in LCA database

Added sugars (in both solid and liquid form) are used in multiple foods and beverages within the LCA database. The replacement criteria are summarized in Table 1, as a function of the added sugar form, or primary bulking ingredient with which the sugar was mixed. For example, in yogurts, milk was assumed to be the main ingredient, which would replace any removed sugar.

**Table 1:** Replacement methods for the various sugars in different foodstuffs, and mass of each ingredient needed to match both replaced mass and relative sweetness of 1 kg sugar

Foodstuff example	Sugar type	GWP of sugar (kgCO_2_-eq/kg)	Replacement	Mass RA60 (g)	Mass bulking agent (g)	GWP of sugar replacement (kgCO_2_-eq/kg)
Granulated sugar	Sugar from beet	0.93	RA60	4.00	–	0.08
Solid foods	Sugar from beet	0.93	RA60 + sorbitol	1.60	998.40^b^	1.63
Sweetened drink	Sugar from beet	0.93	RA60 + water	4.00	996.00	0.08
Yoghurt with fruit, or milk drink^a^	Sugar from beet	0.93	RA60 + milk	3.95	996.05	0.99
Croissant	HFCS	0.52	RA60 + maltitol-BPS	1.20	998.80	0.17
Bouillon cube	Glucose syrup, 32% DM (corn)	0.18	Maltitol-BPS	–	1000.00	0.14

^a^Ingredients are included prior to fermentation in the LCI database, hence, milk is the common ingredient

^b^There are dietary challenges with replacing sugar with sorbitol on a 1:1 basis. These are discussed in Sect.  4.3

In all instances, both the mass of sugar (or syrup) replaced and relative sweetness were considered. It is not possible to replace added sugar directly with RA60 because the high intensity sweetness of the latter means that approx. 4 g is needed to replace 1 kg added sugar [12]. This is not feasible for a foodstuff requiring the bulk (or volume) of sugar. Likewise, sorbitol is only 0.6-times as sweet as sugar [21]. Therefore, an additional NNS is required to make the sorbitol taste as sweet as the sugar it replaces.

The appropriate ratio of RA60 and bulking agent was calculated using Eq. 1, where *RS*_*sugar*_ is the relative sweetness of sugar, and *RS*_*RA60*_ that of RA60, and *RS*_*i*_ the bulking agent.

1 $$\:Mass\:RA60\:\left(in\:kg\right)=\frac{\left({RS}_{sugar}-{RS}_{i}\right)}{\left({RS}_{RA60}-{RS}_{i}\right)}$$

Relative sweetnesses of the other ingredients compared to sugar were assumed to be: 250 for RA60 [12]; 0.7 for maltitol-BPS; 1 for high fructose corn syrup (HFCS), and 0.7 for glucose syrup [40] (the same as maltitol-BPS). For milk, lactose was assumed to have a relative sweetness of 0.2 [41]. It was further assumed that the lactose content of milk is 63.0 g/kg_milk_. This results in an estimated relative sweetness of 0.0126 for one litre of milk. Water has no relative sweetness. The required masses of RA60 and bulking agent in each instance are given in Table 1.

### Diet scenarios

#### Baseline (Sugar) diet

The baseline diet is as observed, with the amount of added sugar derived from the Blonk Food Database LCI (as described above).

#### NNS diet

The NNS diet is the same as the baseline diet, except sugar was replaced by NNS and bulking agents, and the total energy, total carbohydrate, and total mono- and disaccharides per 100 g were recalculated based on the replacements made. Replacement of sugar with NNS in the LCIs was automated in LCA software SimaPro, where 1 kg sugar process was replaced with a 1 kg mix of NNS and bulking agent. The exception was table sugar, for which 1 kg sugar was replaced by 4 g RA60. Table 1 shows an example of food products or types, sugar ingredients, NNS and bulking agent replacement processes used, and GWP of the processes.

### Diet optimization

Diet optimization was conducted using Optimeal 3.0^®^ software to explore differences in dietary changes needed to meet nutritionally adequate and low carbon diets between baseline and NNS diets. In particular, quadratic programming was conducted applying nutritional and environmental constraints in a stepwise manner while minimizing deviation from the starting diet.

The first optimization aligned the diets with recommended nutrient and energy intakes. Constraints were derived from the Dutch population reference intakes, where available, or adequate intakes, as defined by the Health Council of the Netherlands [42–44]. In the Netherlands, there are no recommendations for total daily amount of free sugar consumed, so no constraints were placed on sugar intake. The energy intake constraint was based on the estimated intake of the NNS diet and derived from average calorie intakes of the higher-energy consuming subset in the NNS scenario. Upper limits on nutrients were based on values for tolerable upper intake levels [45]. The full list of upper and lower limits is given in Online Resource Table S4.

For the second optimization, in addition to the nutrient constraints, an upper limit was placed on GWP equal to the 2030 food system greenhouse gas emissions target of 2.04 kgCO_2_-eq/person/day, which was calculated in a previous study based on the IPCC 1.5 °C assessment study [46].

To prevent extremely large changes for single food products, an upper limit was placed on the quantity of each food product equal to the 95th percentile of consumption during each optimization step. Deviation from the baseline to the optimized diets was measured as the Euclidean distance to determine the effort needed to achieve the requirements. Diets with a smaller Euclidean distance were considered easier to achieve [47].

## Results

### Energy and nutrient intake across the whole of each diet

**Fig. 1:**
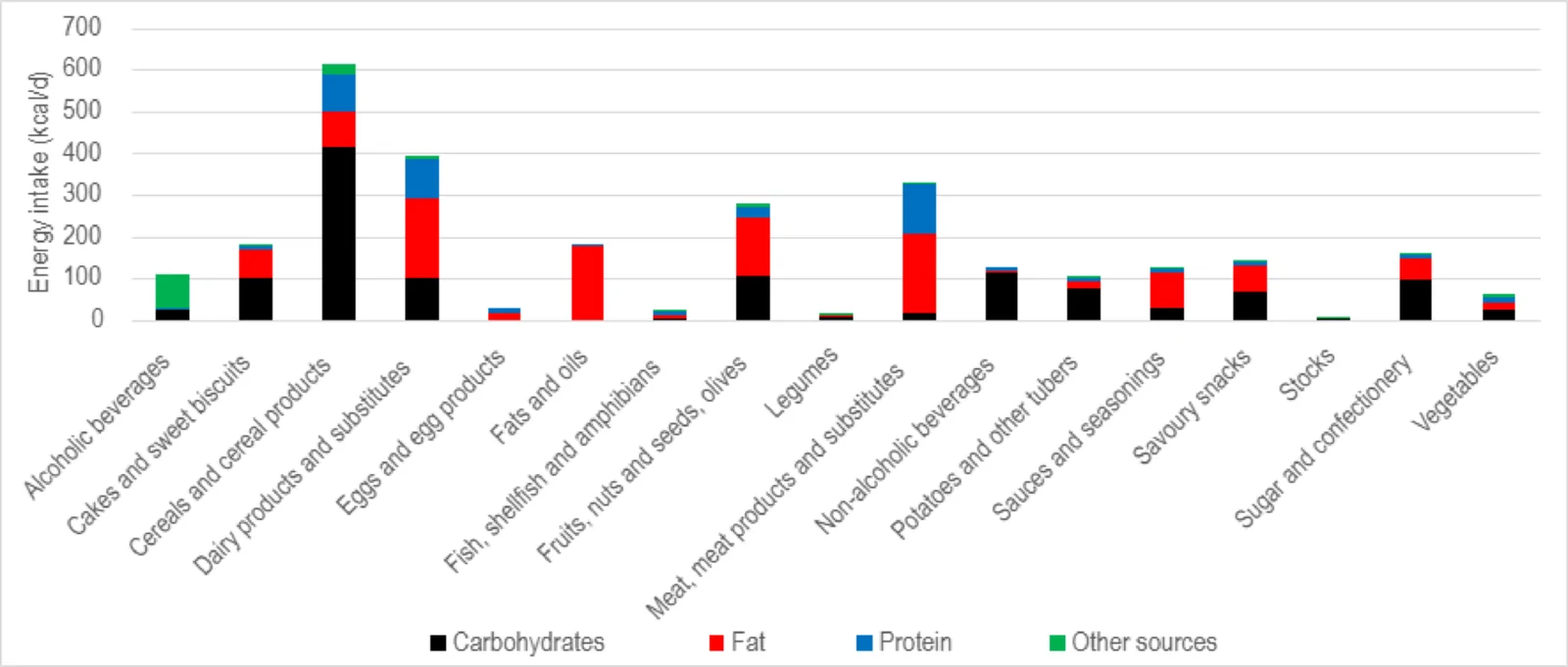
Energy intake in kcal/d as a function of food group and macronutrient. Energy intake shown for carbohydrates (black), fats (red), protein (blue), and other sources (green)

The mean total energy intake of Dutch adults consuming > 2200 kcal/d was 2866 kcal/d, of which 40% comes from fats, 15% from protein, 41% from carbohydrates, 2% from alcohol, and 2% from other sources (e.g., dietary fiber, organic acids). Full nutrient values are given in Online Resource Table S4. The estimated amount of added sugar was 54.1 g/d (8% of total energy intake). Distributions of macronutrients are shown in Fig. 1 across all the food groups included in the study. Energy intake is shown as a function of carbohydrates (black), fat (red), protein (blue), and other sources (green). Full numerical values are given in Online Resource Table S5.

Carbohydrates in the context of Fig. 1 includes mono- and disaccharides (sugars, with no distinction made between naturally occurring, free or added), polysaccharides (starch) and polyols (sugar alcohol). Therefore, Table 2 shows the specific contribution of mono- and disaccharides (total sugars), estimated amounts of added sugar across the food groups for which they were recorded (i.e., vegetables are not in the table), and fractions of added sugar to total sugars (fourth column) and total diet (fifth column). Hence, this shows the opportunity where NNS can be used to replace kcal within the reference diet.

**Table 2:** Contribution of food groups to daily intake (g/d), total mono- and disaccharides, and estimated added sugars. Values include percent of added sugar relative to total mono- and disaccharides within each food group and across the entire diet. Contribution of food group to energy intake is shown in Fig. 1

Food group^a^	Total intake (g/d)	Total mono- and disaccharides (g/d)	Estimated added sugar^b^ (g/d)	% added sugar (relative to total mono- and disaccharides of food group)	% added sugar (relative to entire diet)
Alcoholic beverages	180.7	2.4	0.4	16%	1%
Cakes and sweet biscuits	51.3	13.9	10.6	76%	20%
Cereals and cereal products	266.0	5.8	1.9	32%	3%
Dairy products and substitutes	435.7	21.1	3.8	19%	7%
Fruits, nuts and seeds, olives	172.3	21.0	0.4	2%	1%
Meat, meat products and substitutes	130.5	0.8	0.2	28%	0%
Non-alcoholic beverages	2124.4	26.9	15.8	59%	29%
Sauces and seasonings	53.8	4.9	1.8	37%	3%
Savory snacks	38.7	1.1	0.3	23%	0%
Stocks	15.3	0.1	0.1	90%	0%
Sugar and confectionery	37.2	22.2	18.8	84%	35%

^a^ Food groups without added sugar are omitted. ^b^ Estimated amount of added sugar was derived from the life cycle inventory database

**Fig. 2:**
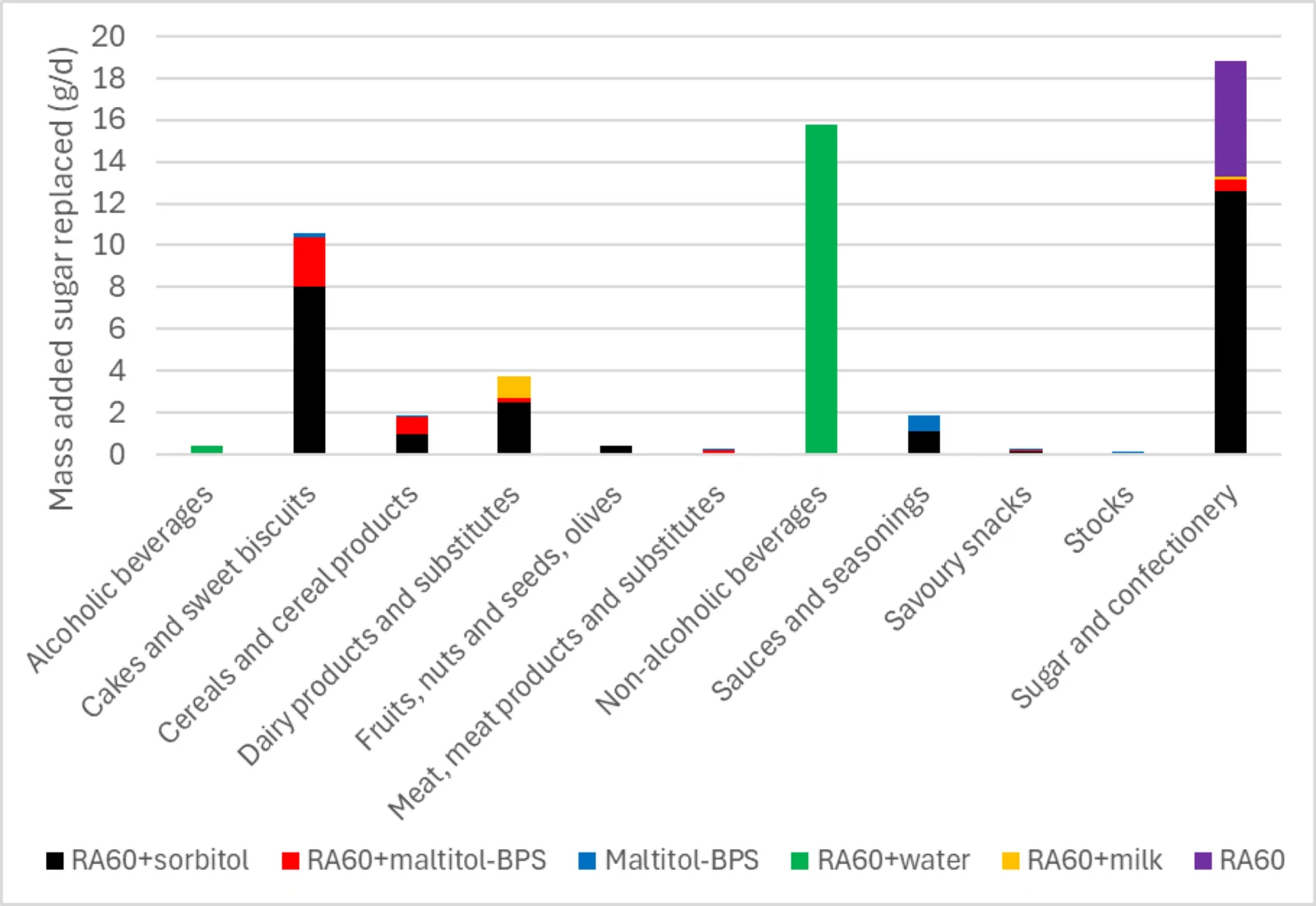
Replacements for added sugar across different food groups. Data shown as a function of mass added sugar replaced. Replacement shown are RA60 + sorbitol (black, replacing granulated sugar in solid foods), RA60 + maltiol-BPS (red, HFCS), Maltitol-BPS (blue, glucose syrup), RA60 + water (green, sugar in drinks), RA60 + milk (orange, sugar in milk-based products), and RA60 (purple, table sugar)

In terms of the NNS diet, total energy intake was 2728 kcal/day, a reduction of 5% compared to the baseline diet. Of this, 41% was from fats, 15% from protein, 40% from carbohydrates, 2% from alcohol, and 2% from other sources. If shown in a graph, the distribution is very similar to that shown in Fig. 1, because the 5% reduction was distributed across multiple food groups. Therefore, instead of reproducing the equivalent graph, full numerical data are given only in Online Resource Table S5. Total carbohydrate consumption reduced from 296 g/d to 276 g/d (6%), and total mono- or disaccharide intake reduced from 125 g/d to 71 g/d (43%). Total added sugar was reduced from 54 g/d to 0 g/d. The following mass of added sugar was replaced by RA60 and/or bulking agent: 25.8 g RA60 + sorbitol; 16.1 g RA60 + water; 5.53 g RA60; 4.1 g RA60 + maltitol-BPS; 1.2 g maltitol-BPS; and 1.2 g RA60 + milk. The distribution of added sugar mass replaced across each food group as a function of what was used to replace it is shown in Fig. 2. Numerical data are given in Online Resource Table S6. The types of sugar replaced are listed in Table 1. For RA60 alone, the actual mass put back was lower than the 5.53 g added sugar removed (purple), because only ~ 22 mg RA60 was used. For all other replacements, it was on a like-for-like mass basis.

**Fig. 3:**
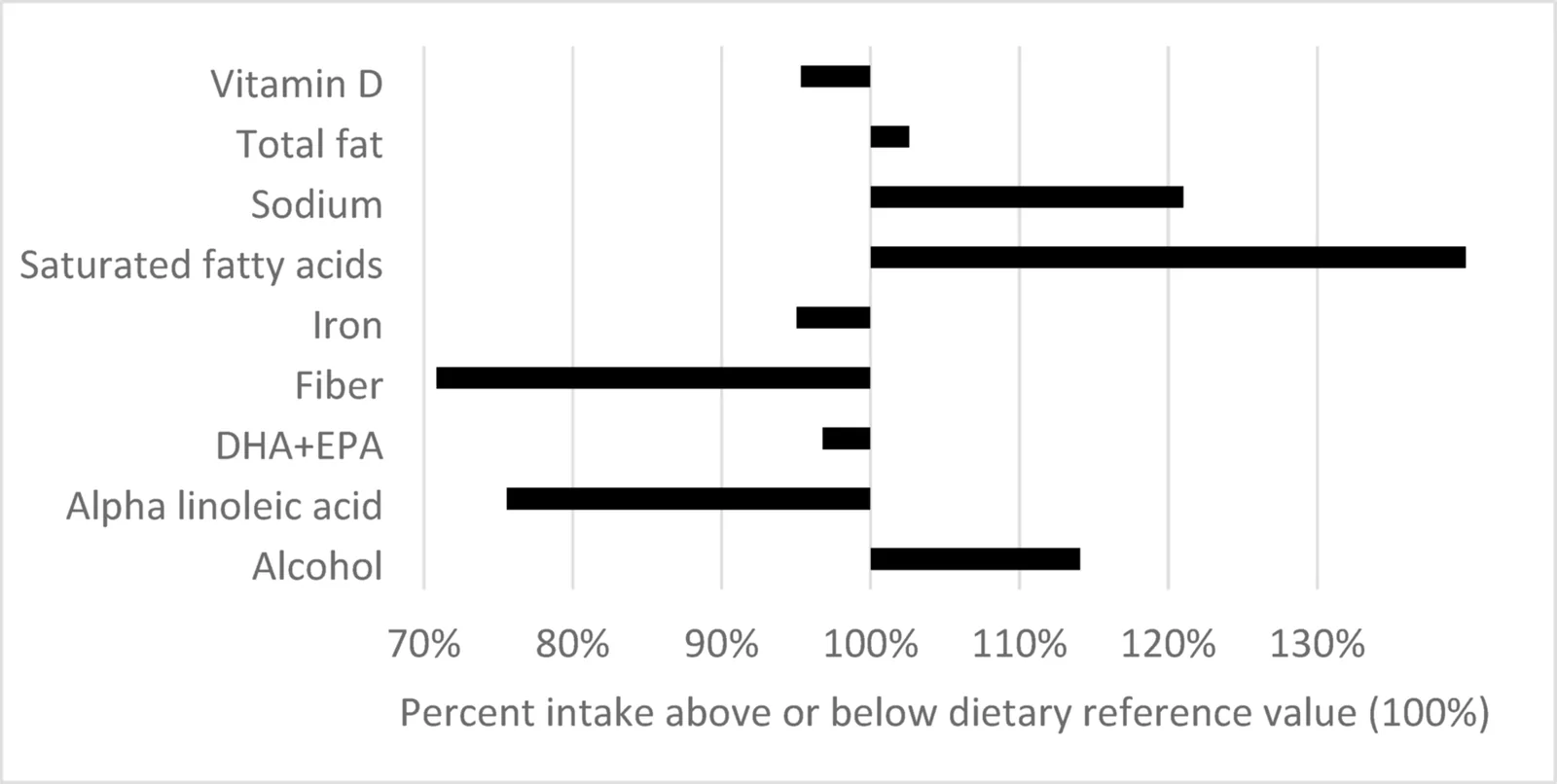
Intake of key nutrients in both NNS and baseline diets above or below dietary reference value (DRV), expressed as a percentage of DRV

Aside from energy, intakes of essential nutrients were identical between the baseline and NNS diets, as only calories were replaced, and no other formulation changes were made. Relative intakes of nutrients in both diets, above and below the dietary reference value (DRV), are shown in Fig. 3. The diets were high in sodium (121% higher than recommended limit), saturated fatty acids (140% higher), and alcohol (114% higher), and low in fiber (71% DRV), alpha linoleic acid (76% DRV), iron (95% DRV), vitamin D (95% DRV), and DHA + EPA (97% DRV). Further optimizations were performed accounting for these DRVs as well as other essential nutrients.

Full dietary intake data for both diets are given in Online Resources Table S4 for baseline nutrient intakes and Table S7 for baseline food intakes.

### Environmental impact change

The environmental impact change from replacing all added sugar is shown in Table 3. The baseline diet had a daily GWP of 4.18 kgCO_2_-eq, land use of 4.46 m^2^a, TA of 1.87 × 10^− 2^ kgSO_2_-eq, FEu impact of 1.82 × 10^− 3^ kgP-eq, MEu impact of 5.88 × 10^− 3^ kgN-eq, and WC of 1.19 × 10^− 1^ m^3^. Similarly, the NNS diet had a daily GWP of 4.18 kgCO_2_-eq. (0% change), land use of 4.46 m^2^a (0%), TA of 1.86 × 10^− 2^ kgSO_2_-eq (− 1%), FEu impact of 1.80 × 10^− 3^ kgP-eq (− 1%), MEu impact of 5.71 × 10^− 3^ kgN-eq (− 3%), and WC of 1.33 × 10^− 1^ m^3^ (+ 11%).

**Table 3:** Total daily environmental impact of baseline and NNS diets, and relative change in impact

Impact category	Units	Baseline diet impact	NNS diet impact	Relative change from baseline to NNS diet (%)
Global warming potential	kgCO_2_-eq/d	4.18	4.18	0
Land use	m^2^a/d	4.46	4.46	0
Terrestrial acidification	kgSO_2_− eq/d	1.87 × 10^− 2^	1.86 × 10^− 2^	− 1
Freshwater eutrophication	kgP-eq/d	1.82 × 10^− 3^	1.80 × 10^− 3^	− 1
Marine eutrophication	kgN-eq/d	5.88 × 10^− 3^	5.71 × 10^− 3^	− 3
Water consumption	m^3^/d	1.19 × 10^− 1^	1.33 × 10^− 1^	+ 11

**Fig. 4:**
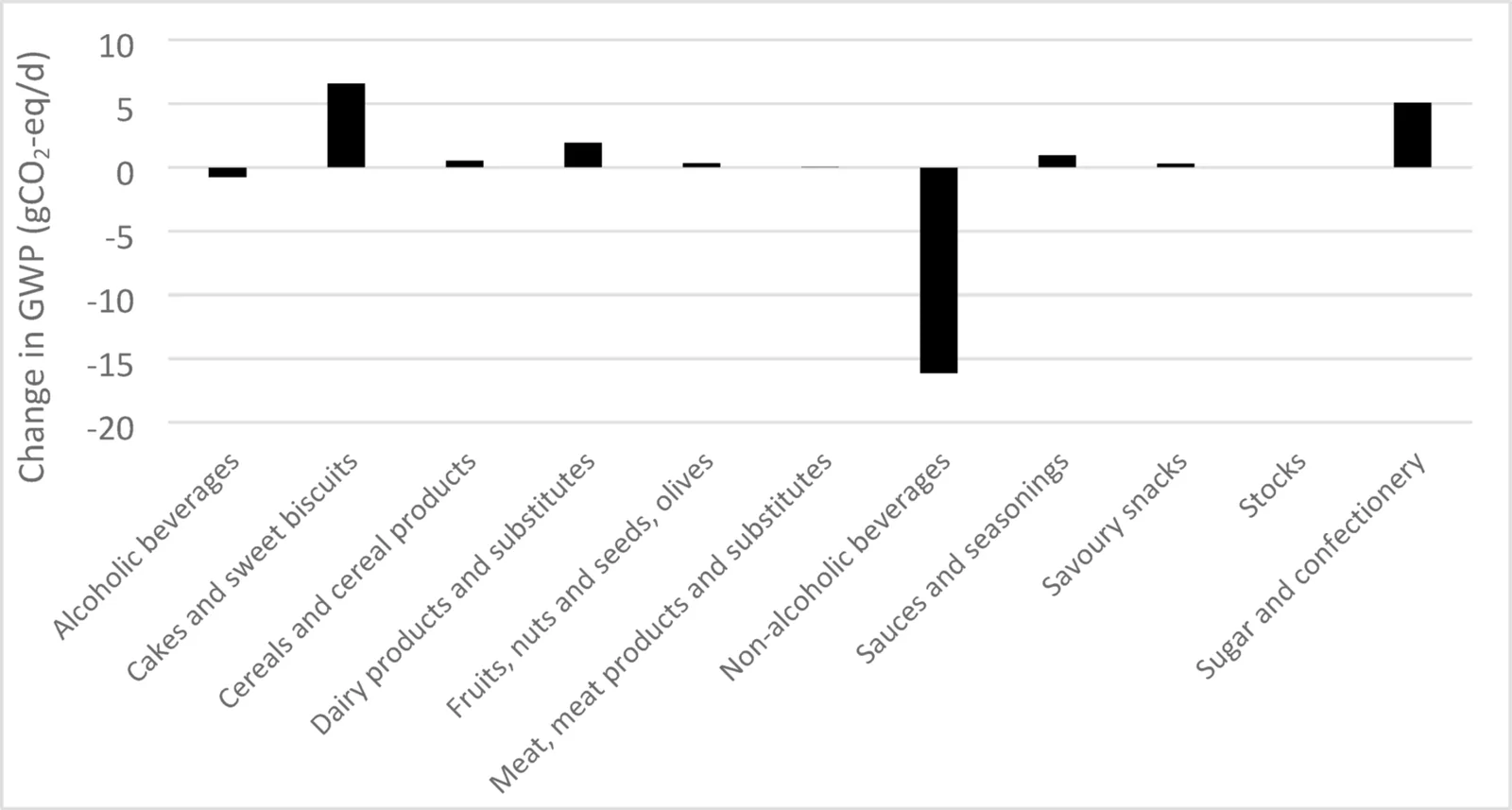
Change in global warming potential between the baseline and NNS diets as a function of food group

While there was no net change in GWP when looking at the whole diet, that of individual food groups did change when RA60 replaced sugar. The change in each individual food group is shown in Fig. 4. Numerical data are given in Online Resource Table S8. Numerical data and graphs for the other impact categories are also provided in Table S8. For GWP, reductions were seen for non-alcoholic beverages (− 16.1 gCO_2_-eq/d) and alcoholic beverages (− 0.77 gCO_2_-eq/d). The reduction occurs because the combination of RA60 and water has a lower GWP than the sugar they replace. This trend is also observed across the other impact categories, except for WC, for which impact increases for non-alcoholic beverages (0.17 l/d). For GWP, the biggest increases were observed in cakes and sweet biscuits (6.6 gCO_2_-eq/d), and sugar and confectionary (5.1 gCO_2_-eq/d). This is primarily due to the RA60 and sorbitol combination having a greater GWP than the sugar they replace. In general, differences in environmental impacts between food groups derive from the different bulking agents used. For instance, the WC of sugar and confectionary increased by 6.6 l/d as the primary replacement was RA60 and sorbitol. In contrast, MEu was the only impact category for which the trends for all food groups were the same (a reduction in impact).

### Environmental impact of optimized diets

Table 4 shows general changes in diets as a result of each optimization. In each case, the daily calorie intake is the same as the baseline NNS diet. When optimizing for nutrient intakes only, both diets show a reduction from their respective baselines for GWP (13–14%) to about 3.65 kgCO_2_-eq/d. There is also reduction in land use (7–9%), TA (15–16%), FEu (9–12%), and MEu (16–18%). However, there is an increase in water consumption (WC) between 3% and 9%. When optimizing for the further constraint of the IPCC 2030 climate target, reductions become more marked for all impact categories. This is most apparent for WC, which - in contrast to the increase observed when optimizing only for nutrition - now shows a reduction of 11% for the sugar diet, and 15% for the NNS diet. When optimizing for GHG emissions, meat consumption is reduced, leading to a net reduction in WC. In contrast, when only optimizing for nutrition, legume intake is increased, which causes the smaller observed increase in WC.

**Table 4:** Energy and carbohydrate intake and environmental impact for sugar and NNS diets when optimized for nutrition only (left columns), and additional GHG constraints (right columns)

	Nutritionally adequate		Meets 2030 climate target	
	Sugar diet (relative change^a^)	NNS diet (relative change^a^)	Sugar diet (relative change^a^)	NNS diet (relative change^a^)
Energy (kcal/d)	2728 (− 5%)	2728 (0%)	2728 (− 5%)	2728 (0%)
Total carbohydrates (g/d)	305 (+ 3%)	304 (+ 10%)	308 (+ 4%)	298 (+ 8%)
Mono- and disaccharides (g/d)	115 (− 8%)	75 (+ 5%)	111 (− 11%)	72 (+ 1%)
Total added sugar (g/d)	43 (− 20%)	0 (0%)	43 (− 20%)	0 (0%)
Carbon footprint (kgCO_2_-eq/d)	3.61 (− 14%)	3.65 (− 13%)	2.04 (− 51%)	2.04 (− 51%)
Land use (m^2^a/d)	4.06 (− 9%)	4.16 (− 7%)	2.68 (− 40%)	3.09 (− 31%)
Terrestrial acidification (kgSO₂-eq/d)	1.58 × 10^− 2^ (− 16%)	1.59 × 10^− 2^ (− 15%)	8.83 × 10^− 3^ (− 53%)	8.78 × 10^− 3^ (− 53%)
Freshwater eutrophication (kgP-eq/d)	1.61 × 10^− 3^ (− 12%)	1.64 × 10^− 3^ (− 9%)	1.06 × 10^− 3^ (− 42%)	1.05 × 10^− 3^ (− 42%)
Marine eutrophication (kgN-eq/d)	4.85 × 10^− 3^ (− 18%)	4.80 × 10^− 3^ (− 16%)	2.67 × 10^− 3^ (− 55%)	2.58 × 10^− 3^ (− 55%)
Water consumption (m^3^/d)	1.22 × 10^− 1^ (+ 3%)	1.45 × 10^− 1^ (+ 9%)	1.06 × 10^− 1^ (− 11%)	1.13 × 10^− 1^ (− 15%)
Euclidean distance	29	28	58	60

^a^ Percentage changes from the baseline diets are given in brackets below each environmental impact value

### Change in consumption patterns following optimization

**Fig. 5:**
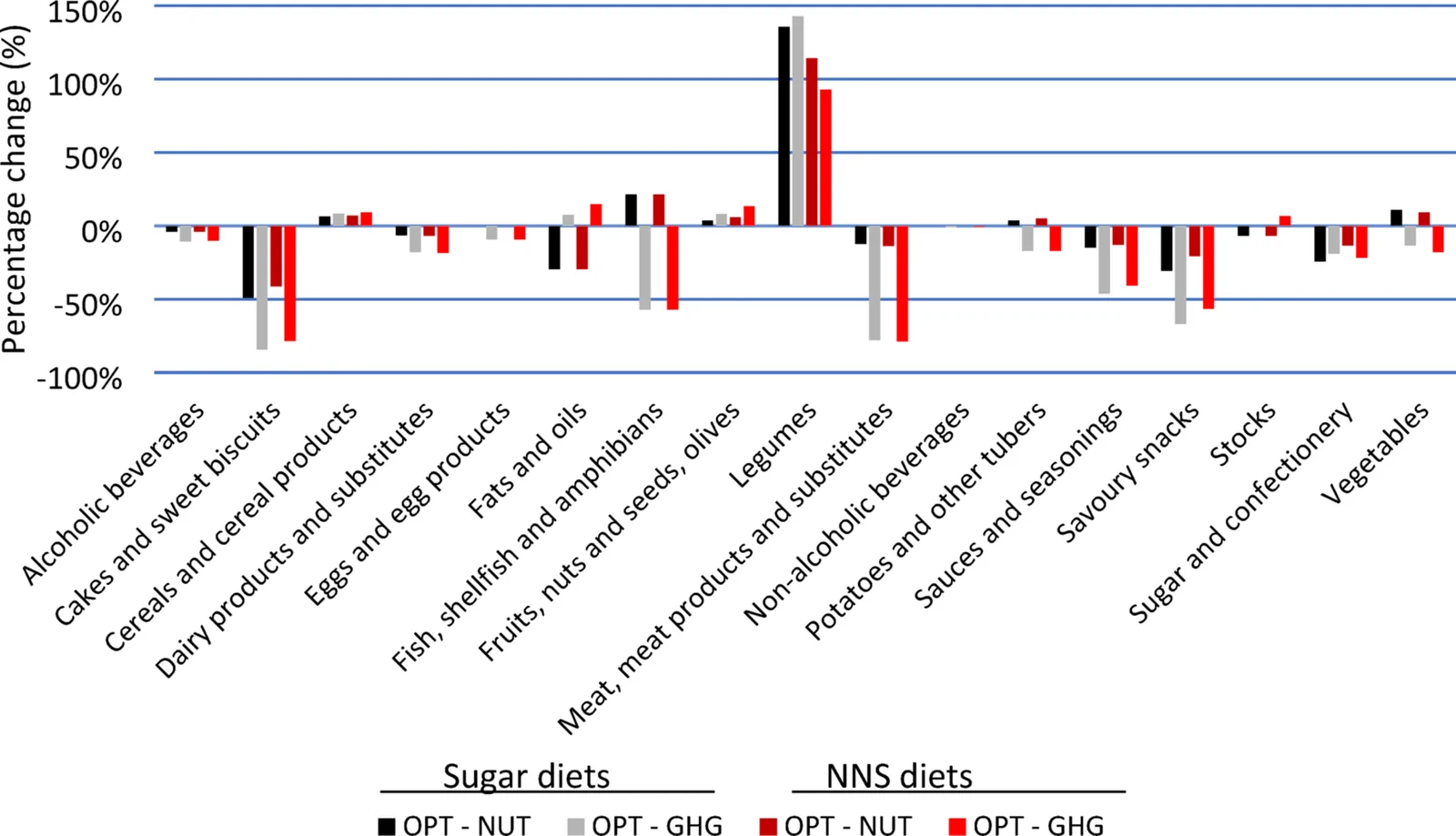
Composition of diets optimized for nutritional adequacy (OPT-NUT) and for nutrients plus 2030 climate target (OPT-GHG) relative to sugar and NNS starting diets

In this section, details of the changes in consumption of different food groups are explored by way of understanding the change in environmental impacts. Figure 5 shows the percentage change in intake (in grams per day) of food groups between the sugar and NNS starting diets and both optimized diets. Relative changes in consumption for diets optimized for nutrient intake are shown for sugar diets (black), and NNS diets (dark red). Numerical data are given for both absolute masses consumed and relative change from baseline in Online Resource Table S9. With respect to the nutrient-focused optimization, the directions of changes in intake of food groups were similar for both diets, but the magnitude of changes differed. For instance, cakes and sweet biscuits, savory snacks, and sugar and confectionary reduced in both diets, but to a lesser extent in the NNS diet (equivalent to an extra 2 cookies, handful of chips, and half of standard-sized chocolate bar a week). While intake of fruits, nuts, and seeds increased in both diets, it was slightly higher in the NNS diet, specifically due to the inclusion of more nuts (equivalent to an extra small handful of nuts a week). Legumes more than doubled in both diets, but the sugar diet required slightly more (equivalent to an extra quarter cup of cooked lentils). The increase in legumes and nuts contributed to increases in alpha linoleic acid, iron, fiber, and other nutrients. However, as legumes have a lower energy density compared to nuts, legumes were favored in the sugar diet, as energy was a binding constraint, while nuts were favored in the NNS diet. The increase in legume consumption was also a significant contributor to the increased WC impact shown in Table 4. Despite these differences, the Euclidean distance was nearly identical between the sugar and sweetener diets (16.58 vs. 16.22, respectively), meaning the same effort is needed to meet nutrient requirements regardless of the starting diet.

Also shown in Fig. 5 are the changes in consumption (in grams) relative to the starting diets of food groups for sugar diets (grey) and NNS diets (light red). Compared to optimizing only for nutrition, when adding an upper constraint on GHG emissions, a change in direction for intake occurs for a few food groups, such as fish and vegetables, which now decrease in quantity relative to baseline, and fats and oils, which now increase relative to baseline. The reduction in meat consumption is greater for both diets in this optimization regime. This is a significant cause of the reversal in WC impact from the nutrition-only optimization. Despite differences seen between the relative changes in the sugar and NNS diet, the Euclidean distance was again nearly identical (42.31 vs. 42.50, respectively). Again, this means that the same effort is needed to simultaneously reduce carbon footprint and meet nutrient requirements, regardless of starting diet.

## Discussion

In this section, discussion points arising from the results are made, before the challenges are described in the following section.

### Overall change in environmental impact

The environmental impact of the NNS diet was relatively unchanged from the baseline. This is because any reduction in environmental impact achieved by replacing added sugar in water-based drinks, is offset by an increased impact from using sorbitol as a replacement in solid formulations. This indicates potential for a future reduction in environmental impact with more careful re-formulation of food products. For instance, when replacing added sugar in water-based drinks, there is little option but to use water. However, for solid foods, sorbitol is unlikely to be the best replacement in all circumstances, with an opportunity to use alternative bulking agents with lower environmental impacts.

### Choice of background LCA data

The change in environmental impact when replacing ingredients is dependent on the choice of ingredient and background LCA. The added sugar in this study was all derived from beet; used here because of the study’s European focus, and dominance of beet production in the EU [48]. However, cane sugar makes up approx. 80% of world sugar supply [49]. The environmental impact of cane sugar *tends* to be greater in GWP, TA, FEu and WC, lower for MEu, and highly dependent on production location for land use.

For sorbitol, another study reported greater values for GWP (2.2 kgCO_2_-eq/kg) and TA (14.2 × 10^− 3^ kgSO_2_-eq/kg) than those used here, with the other impact categories not reported [25].

This highlights how choice of background data could potentially influence the results. For instance, if only cane sugar were considered, then environmental impact of the NNS diet *might* have been lower across all impact categories.

### Dietary issues

When added sugars were replaced in the diet, it required 25.8 g of sorbitol, 5.3 g of maltitol-BPS and 0.7 g of lactose (in the milk) per day. These are considered FODMAPS (fermentable oligosaccharides, disaccharides, monosaccharides and polyols) and hence may cause problem for some 18–22 million people in Europe who meet criteria associated with FODMAP sensitivity [50].

Likewise, across the entire diet, 138.5 mg of RA60 was required to replace sugar. The maximum acceptable daily intake (ADI) is set at 4 mg steviol glycoside equivalents/kg body mass and not mg of stevia [51]. In context, 138.5 mg of RA60 represents roughly 54.3 mg steviol equivalents per day, which is approximately about 18% of the ADI for a 75 kg person. Therefore, it is unlikely that consumption of RA60 will be in excess, but it is still at a level that would require monitoring if considering such wholesale replacement of added sugar.

Relative to other NNS, RA60 has one of the lowest sweetness equivalencies, and one of the lowest ADIs [52]. This is encouraging as it indicates that any other NNS will have a greater margin of intake when replacing added sugar in the same manner as this study.

### Realism of added sugar replacement

This study did not include impacts from consumption (including changes in health) or wastewater treatment of any of the diets. A report from the World Health Organization (WHO) concluded that there were not significant changes to health from consuming NNS in place of added sugar [9]. Until more conclusive studies are published, however, it is not feasible to extend environmental impact to include effects of health changes, and the scale of subsequent health interventions.

It is unlikely that any diet would replace all added sugar. Therefore, this study is purely theoretical in that regard. By replacing all added sugar, this study shows the potential maximum range of environmental changes that might occur. If sugar were partially replaced across all foods, the change in environmental impact might be expected to lie somewhere between the extremes. However, if added sugar were replaced only in certain food groups, Fig. 4 (and the graphs in Table S8) gives insight into where there is greatest opportunity to reduce impact (e.g., GWP in drinks), and which foods risk increasing impacts (e.g., GWP for sugar and confectionary, and cakes and sweet biscuits). It would be better to seek changes that are sensitive to the local needs, individuals, locations, or pre-existing diets. For instance, it is not necessarily the recommendation that *all* added sugar is removed from a diet, but simply that consumption is reduced. Likewise, for instances of overconsumption of daily calories, alternative solutions may be sought in dietary change, instead of simply replacing added sugar.

### Assumptions and future research recommendations

The final objective of the study was to explore and catalogue challenges associated with undertaking such research. In this section, issues encountered, and future research areas, are outlined, which may help overcome some of the challenges. First and foremost, the authors acknowledge that the challenges have impacted the specific results of the study, and that refinement of the methodology and underlying data will change the results. Secondly, some assumptions were by necessity broad, such as rules for replacing which added sugar in what foods. The methodological choice was made to use fewer simple, well-defined assumptions, rather than multiple, nuanced, unsupported assumptions.

#### Background dietary data

The first challenge relates to identifying a representative diet and linking that diet to LCA databases. There is no such thing as a “representative” European diet, which spans multiple countries, regions, ages or body types. Similarly, diets include a diverse range of foodstuffs, not all of which have formulation data available, or with all ingredients available in an LCA database. Some foods are only consumed in very small quantities. Those contributing < 1% of mass or calorie intake were omitted, including meal replacers, and in all cases, mapping was needed to bridge data gaps (as outlined in Sect.  2.1). Unrepresented foods were accounted for by redistributing mass within the food group. In addition, where ingredient data were not available in the LCI, proxies were used. Changes in any of these will affect the results of this study. However, the diet chosen for this study was forced to one assumed to be high in added sugar. It would have been preferable to select diets using a constraint for those with > 10% calories from added sugar. However, added sugar is not recorded in DNFCS. Therefore, overconsumption of calories was used as a proxy for overconsumption of added sugar, as they tend to correlate in Western diets [53]. Diets with less added sugar would likely have smaller change in environmental impact (when substituting in the NNS) due to the smaller mass of sugar compared to other energy-producing ingredients.

The original intent was to select a diet using BMI as the criteria. However, energy intake was found to be underreported compared to the average Dutch diet. Therefore, the selection was forced for diets with high energy intake. This was assumed to equate to an unhealthier diet, but it might also be equally attributable to a highly active person’s diet. More refined data into reported energy intake, and BMI would be needed to choose more appropriate diets for this kind of study.

#### Estimating added sugar

Added sugars are not chemically different from naturally occurring sugars and thus are not measured analytically. While methods have been developed to estimate added sugar content e.g., [54, 55], this study took a pragmatic approach and relied on general recipe and ingredient information available in the Blonk Food Database LCIs and total amounts of sugar in the Dutch Food Composition Database (NEVO). In several instances, the estimated amounts of added sugar were greater than total sugar. In such cases, the amount of added sugar was capped at the amount of total sugar in NEVO. This issue was outlined in Sect.  2.3.1. However, there were instances where it was the other way round. In this instance, we relied fully on the LCI recipe data to estimate amounts of added sugar. In a previous study, the estimated median added sugar intake was 64 g/d among the Dutch population, or 12% of total energy intake [2]. While total sugar intake has decreased over the years [56], our approach to estimating added sugar in the diet may have underestimated dietary added sugar intakes, as we measured an average intake of 8% of total energy intake. Given the importance of this single data point to the whole study, it is an area for further research, or an opportunity to include individual sugars in national food composition databases.

Related to this, there was often not enough nuance in formulation data. Aside from issues of mapping, it was not possible to capture differences that occur between brands for otherwise similar products. For some foodstuffs, these might be similar (e.g., crisps), but for others (e.g., biscuits or cakes), differences may be more marked. Future studies would benefit from a stronger link between brand variability in formulation, and links to food composition and LCA databases.

#### Replacing sugar by reformulation

In all instances where added sugar was found, it was replaced by RA60 and a bulking agent. This is already known to be an inaccurate assumption. For instance, it has been shown that yogurt can be reformulated using existing ingredients to account for removal of added sugar [18]. In this study, we assumed milk was the bulking agent but, in that study, a combination of milk, protein powder and cream were adjusted instead. It is likely that there are multiple instances across the whole diet where reformulation is possible. However, without better reformulation data for every instance, it is not possible to make a better judgement on how to replace the added sugar.

We also assumed that the same bulking agent could be used across a food group. This is unlikely to be correct. For instance, sorbitol was used in many instances, but there will be opportunities to use different ingredients, e.g., for reasons of maintaining a “clean label” ingredients list. Clean label is not well defined, but there will be markets for low added sugar foods in which customers may eschew ingredients like sorbitol.

The only NNS used in this study was RA60. However, the most common ones are sucralose and aspartame. It is not anticipated that changing the NNS will significantly alter the findings of this study, but it is still a nuance in formulation data that should be accounted for where possible.

#### Lack of background data

Lack of background LCA data will be a significant barrier to progressing research in this area. For instance, there are many other sugar alcohols, such as xylitol or erythritol. At the time of the study, the only other sugar alcohol with LCA data was maltitol. Therefore, even if formulation data using other sugar alcohols were available, sorbitol would still need to be used as a proxy. More research is required to fill this data gap to support flexibility of ingredient replacement.

There is a similar lack of LCA data for NNS. Studies relating to aspartame, sucralose, and neotame were all derived from patent literature, and from first principles for chemical reactions [14, 15]. More studies supported by industry would refine this LCA data. In addition, there are no LCA studies in the public domain for any other NNSs.

Sweetness equivalence is an approximate value, with perceived sweetness depending on formulation or concentration in a foodstuff. Assumed values of 250-times for RA60 and 0.6-times for sorbitol were used, but more refined data relating to sweetness equivalence for any NNS or bulking agent is required to improve the substitutions made.

The Blonk Food Database does not capture where NNS might exist within an existing diet. For instance, many soft drinks consumed already contain NNS instead or alongside added sugar. It was assumed that the baseline diet only included drinks sweetened with added sugar. More data regarding existing NNS intake in baseline diets is needed for future studies. However, regardless of existing NNS consumption, the overarching question of replacing all added sugar with NNS is still valid as environmental impact change will have already occurred from pre-existing replacements in the current diet. Therefore, this study is exploring the extremes of what is possible, which encompasses the real-life situation within its bounds.

## Conclusions

This is the first study in which the environmental ramifications of replacing added sugar with an NNS in a whole diet setting are explored. Of the specified objectives, the three key ones were to identify where sugar could be replaced in a diet, to explore the environmental impacts of doing so, and understand the challenges of undertaking such a study.

Arguably, the main limitation was lack of disaggregation of dietary added sugar in the DNFCS database from total sugar. This study therefore needed to make assumptions regarding food formulation to effectively replace added sugar. If replacing added sugar with NNS should become a policy objective for health reasons, it will be necessary to ensure finer disaggregation of added sugar in food composition databases to support such activities.

Having replaced added sugar with NNS, this study showed that it is possible to replace it all with relatively limited adverse environmental consequences. This important finding demonstrates that, firstly, partial replacement of added sugar will result in even smaller deviations in environmental impact, and secondly, that changes can focus on health outcomes instead. Moreover, the results showed that the easiest place to replace added sugar with NNS, in non-alcoholic beverages, yields the greatest environmental reduction in impact.

When optimizing diets for nutritional and environmental targets, both sugar- and NNS-based diets responded similarly. This is another important finding, as it adds further reassurance that there is a wide range of options for policy making when seeking to improve healthiness and reduce environmental impact of the foods we consume.

In terms of the diets, this study took a pragmatic approach to identifying potentially unhealthy diets; by focusing on high calorie intake. However, it was not clear from the DNFCS database that they were all unhealthy, or whether some were for very active individuals. Therefore, to support further research in this area, it would be beneficial if activity levels were recorded alongside dietary intake.

In summary, there are a range of options to improve dietary and environmental outcomes when seeking to replace added sugar. To make the most of these options, there are several areas in which foods formulation may be explored further to identify the optimal bulking agents. But even if simple steps are taken, the results of this study show there is plenty of room for maneuver to improve both nutrition and environmental impact of the foods we eat.

## Supplementary Information

Below is the link to the electronic supplementary material.

Supplementary Material 1

## Data Availability

All data relating to this research are available in this document and supporting material, with exception of commercially sensitive data, as outlined in the text.
